# Methylation of CpG sites in the upstream regulatory region, physical status and mRNA expression of HPV-6 in adult-onset laryngeal papilloma

**DOI:** 10.18632/oncotarget.19898

**Published:** 2017-08-03

**Authors:** Zeyi Deng, Taro Ikegami, Asanori Kiyuna, Chunlin Zhang, Tao Zhang, Sen Matayoshi, Takayuki Uehara, Hiroyuki Maeda, Mikio Suzuki, Akira Ganaha

**Affiliations:** ^1^ Department of Otorhinolaryngology, Head and Neck Surgery, Zhujiang Hospital, Southern Medical University, Guangzhou, China; ^2^ Department of Otorhinolaryngology, Head and Neck Surgery, Graduate School of Medicine, University of the Ryukyus, Okinawa, Japan; ^3^ Department of Otorhinolaryngology, Head and Neck Surgery, Affiliated Hospital of Zunyi Medical College, Zunyi, China

**Keywords:** adult-onset laryngeal papilloma, human papillomavirus-6, methylation, physical status, mRNA expression

## Abstract

The methylation status of HPV-6 upstream regulatory region (URR) in adult-onset laryngeal papillomatosis (AO-LP) remains unclear. The purpose of this study was to investigate the methylation status of URR and the physical status of HPV-6, as well as the dynamic variations of viral load and mRNA expression in AO-LP. We examined 18 specimens from 11 patients with AO-LP by real-time polymerase chain reaction (PCR), bisulfite-sequencing PCR, and amplification of papilloma oncogene transcripts. HPV-6 was identified in 9 of 11 patients (81.8%), and all the 15 specimens derived from 9 HPV-6-positive cases contained only episomal HPV-6 transcripts with intact *E2*. Three HPV-6-positive patients developed recurrent lesions, and HPV-6 copy numbers and mRNA expression decreased after surgical treatment. Among the 96 CpG sites (16/case), 67 (69.8%) were unmethylated, while 23 (30.2%) were heterogeneous (≥ 1 methylated CpG clone). High viral loads and episomal status of HPV-6 were frequently observed in AO-LP; thus, persistent *E6/E7* mRNA expression of LR-HPV-6 may be associated with AO-LP recurrences. Hypomethylation and scattered patterns of methylated CpGs at the URR of HPV-6 were identified in AO-LP.

## INTRODUCTION

Human papillomaviruses (HPVs) are small icosahedral viruses containing 8 or 9 genes on circular double-stranded DNA. More than 150 types have been identified to date. HPV infection can stimulate benign tumors, promote oncogenic changes, or be asymptomatic in epithelial cells [1]. Adult-onset laryngeal papillomatosis (AO-LP), usually a benign tumor of the larynx, is induced by low-risk human papillomaviruses (LR-HPVs), especially HPV-6 and -11 [2, 3]. AO-LP is characterized by recurrences and warty exophytic lesions in the larynx, but can spread to the whole respiratory tract including the lungs in up to 20% of adult patients [2, 4]. The recurrent nature of AO-LP could be due to persistent viral reservoirs and activation of asymptomatic HPV infections [5]. Malignant transformation can occur, but this has been reported in less than 3% of cases [6, 7].

It is well known that oncogenic HPVs encode two main viral oncoproteins, E6 and E7, which are responsible for the maintenance of HPV-mediated malignant transformation by their interactions with p53 and retinoblastoma (pRb) tumor suppressor proteins, respectively. Unlike high-risk HPVs, E6 and E7 proteins of LR-HPVs have substantially lower transforming activity and do not induce genomic instability. LR-HPV E6 proteins lack the ability to degrade and inactivate p53 [8], while low-risk E7 proteins bind to pRb less efficiently and do not induce pRb destabilization [9]. The viral E2 protein participates in viral DNA replication and transcription and regulates the expression of E6 and E7 oncogenes. Moreover, the activities of E2 protein are related to its ability to bind to 4 conserved E2 binding sites (E2BS) in the upstream regulatory region (URR), especially the early promoter p97 [10, 11].

The methylation status of the HPV-16 genome in cancer cell lines and clinical samples of the cervix, head, and neck has been analyzed and identified as an important feature in malignant transformation [12–16]. Previous studies have reported progressive methylation of URR in HPV-16-infected cervical specimens, and HPV-16 URR methylation rates of 5.9%, 33.3%, and 53.3% in low-grade squamous intraepithelial lesion (SIL), high-grade SIL, and squamous cell carcinoma (SCC), respectively. Moreover, de novo methylation or mutations of E2BSs in the URR might cause upregulation of *E6* and *E7* oncogene expression by impairing the ability of *E2* to bind to its specific sites [12, 17]. In a study by Park et al., 9 of 22 (41%) oropharyngeal SCCs were methylated in more than 50% of the CpG sites at either the *E6* enhancer region or the E2BS [14]. However, few reports have been published to date and knowledge on the methylation of LR-HPVs in AO-LP is limited. Ure and Forslund [18] demonstrated that the URR of HPV-6 is completely unmethylated in papillomas of the aerodigestive tract, which is very different from findings in malignant tissues of the cervix and oropharynx. Nevertheless, the methylation status of the HPV-6 URR in AO-LP was not established because of the relatively limited number of specimens in the previous study. In addition, a high HPV-16 load was found to correlate significantly with *E6/E7* mRNA expression in SCC of the head and neck in our previous study, suggesting that variations in HPV viral load might reflect differences in the capacity of the viral DNA to replicate and its transcriptional activity [19]. With respect to LR-HPV tumorigenesis in AO-LP, the relationship between the dynamic variations among LR-HPV loads and the high rate of AO-LP recurrences remain to be clarified.

In this study, we investigated the methylation status of the URR of LR-HPV-6, the physical status and expression of *E6/E7* mRNA in AO-LP, and the dynamic variations of HPV-6 among primary tumors and recurrent tumors after treatment. Moreover, we analyzed the relationship between the methylation pattern of the HPV-6 URR and viral load in AO-LP.

## RESULTS

### Prevalence of HPV and subtypes in AO-LP

The clinicopathological characteristics of 18 specimens obtained from 11 patients with laryngeal papilloma are summarized in Table 1. All patients underwent surgical treatment, including three cases who underwent at least 2 operations. The mean interval to recurrence was 13.0 months among the 3 cases (case 5, case 6, and case 7). In case 5, the primary lesion was unilateral but the tumor in the second recurrence developed bilaterally in the true vocal cords. The prevalence of HPV in AO-LP was 81.8% (9 of 11), and all HPV-positive samples were infected with the LR-HPV-6 subtype. Only 1 of the 11 patients (case 8) developed laryngeal SCC within 2 years of receiving surgery for laryngeal papilloma; however, HPV DNA was not found in either the malignant or benign lesions in this case.

**Table 1:** Clinicopathological characteristics of 11 patients with AO-LP

Case No.	Age (years)	Sex	Tumor size	Subsite	HPV	Viral load (copies/ng DNA)	mRNA (E6/actin)	APOT	p16	p53	pRb	MT
1	31	M	12 mm	R, TVC	HPV-6b	603,566	0.18	Epi.	+	–	–	N
2	58	F	20 mm	R, AF	–				–	+	+	N
3	27	M	14 mm	R, FVC	HPV-6a	2,268,452	0.08	Epi.	+	–	–	N
4	43	M	7 mm	B, TVC	HPV-6b	676,099	0.26	Epi.	–	–	+	N
5	67	M	12 mm	L, TVC	HPV-6vc	178,463	0.18	Epi.	–	–	+	N
5-R1			10 mm	L, TVC		132,452	0.31					
5-R2			15 mm	B, TVC		182,549	0.32					
5-R3			12mm	B, TVC		28,594						
5-R4			12mm	B, TVC		9,550	0.10					
6	17	F	10 mm	B, TVC	HPV-6vc	31,725	0.08	Epi.	–	–	–	N
6-R1			12 mm	R, TVC		27,756	0.06					
7	59	M	8 mm	B, TVC	HPV-6vc	712,653	0.21	Epi.	–	–	–	N
7-R1			8 mm	B, TVC		266,558	0.15					
8	66	M	20 mm	R, TVC	–				–	–	–	Y
8-R1			18 mm	R, TVC								
9	45	M	10 mm	B, TVC	HPV-6*	ND	ND	Epi.	–	+	+	N
10	41	M	6 mm	R, TVC	HPV-6vc	25,346	0.42	Epi.				N
11	32	M	18 mm	B, TVC B, FVC	HPV-6a	2,933	0.43	Epi.				N

AO-LP, adult-onset laryngeal papillomatosis; HPV, human papillomavirus;

M, male; F, female; Epi, episomal; MT, malignant transformation; N, no; Y, yes;

ND, Not done; APOT, amplification of papillomavirus oncogene transcripts;

R, right; L, left; B, both; TVC, true vocal cord; FVC, false vocal cord; AF, aryepiglottic fold

*The HPV-6 subtype could not be identified due to insufficient tissue.

### HPV-6 viral load, E6 mRNA expression, and dynamic variation pre- and post-operation

The pre-operative viral load in 9 primary laryngeal papilloma samples positive for HPV-6 DNA was first evaluated. The relative HPV-6 viral load was normalized by the β-globin standard curve and adjusted to values per nanogram of input DNA for each sample. The viral load of HPV-6 ranged from 2,933 to 2,268,452 copies/ng, with a median of 391,015 copies/ng. *E6* mRNA transcripts for HPV-6 were detected in all cases positive for HPV-6 DNA, and the median mRNA quantity was 0.20 (range 0.08 to 0.43).

**Figure 1:**
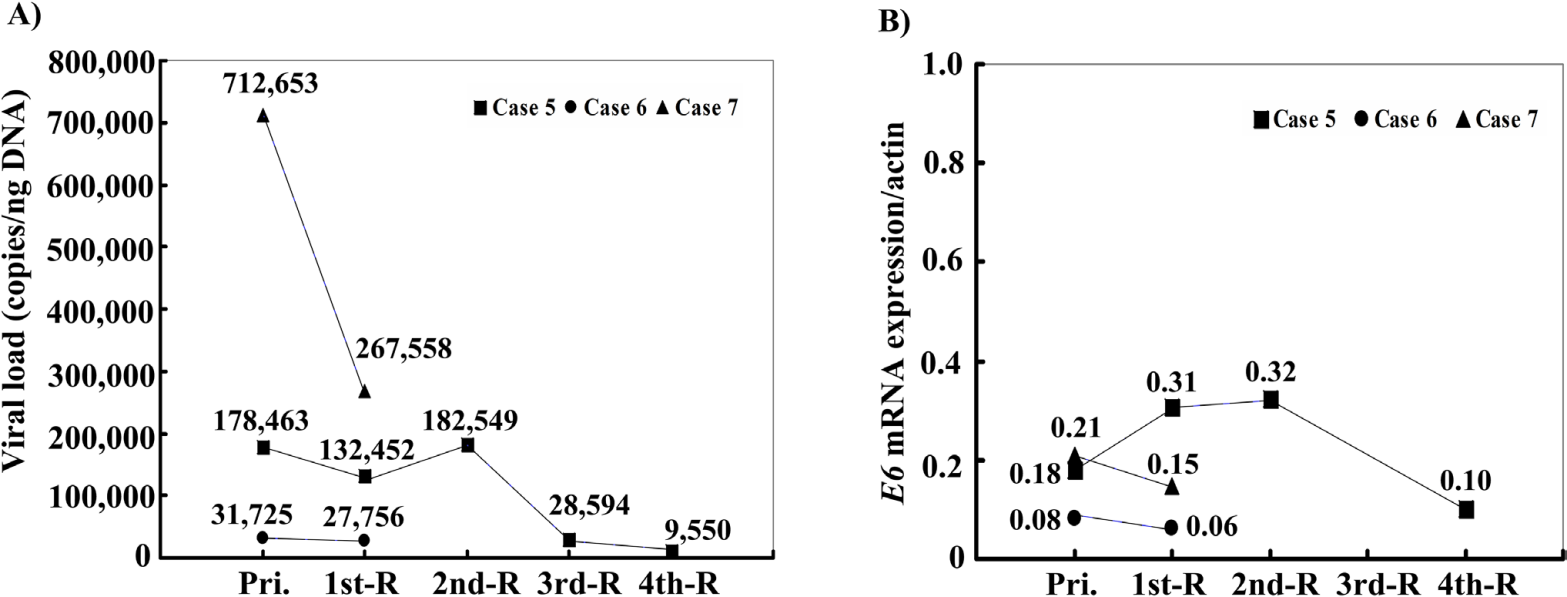
**(A)** Dynamic variation of viral load and **(B)** E6 mRNA expression of HPV-6 in AO-LP before the first treatment, and after recurrences. Compared with the primary lesion, a gradual decline in the tendency of HPV-6 copy numbers and mRNA expression in the recurrent lesions was found in cases 5, 6, and 7 over time after surgical treatment. Pri.: primary tumor without treatment; 1st-R: first recurrence; 2nd-R: second recurrence; 3rd-R: third recurrence; 4th-R: fourth recurrence.

During the observation period, 3 of the 9 HPV-6-positive patients developed recurrent lesions. Viral loads of 712,653 copies/ng in primary lesions in case 7 decreased to 267,558 copies/ng in recurrent lesions (Figure 1A). The expression levels of *E6* mRNA in case 7 slightly decreased from 0.21 to 0.15. Case 5 had 4 recurrences after the primary operation, and the viral load and the expression of E6 mRNA gradually decreased from 178,463 and 0.18, respectively, to 9,550 and 0.10. (Figure 1B).

### Physical status analysis of HPV-6 by amplification of papillomavirus oncogene transcripts assay

**Figure 2:**
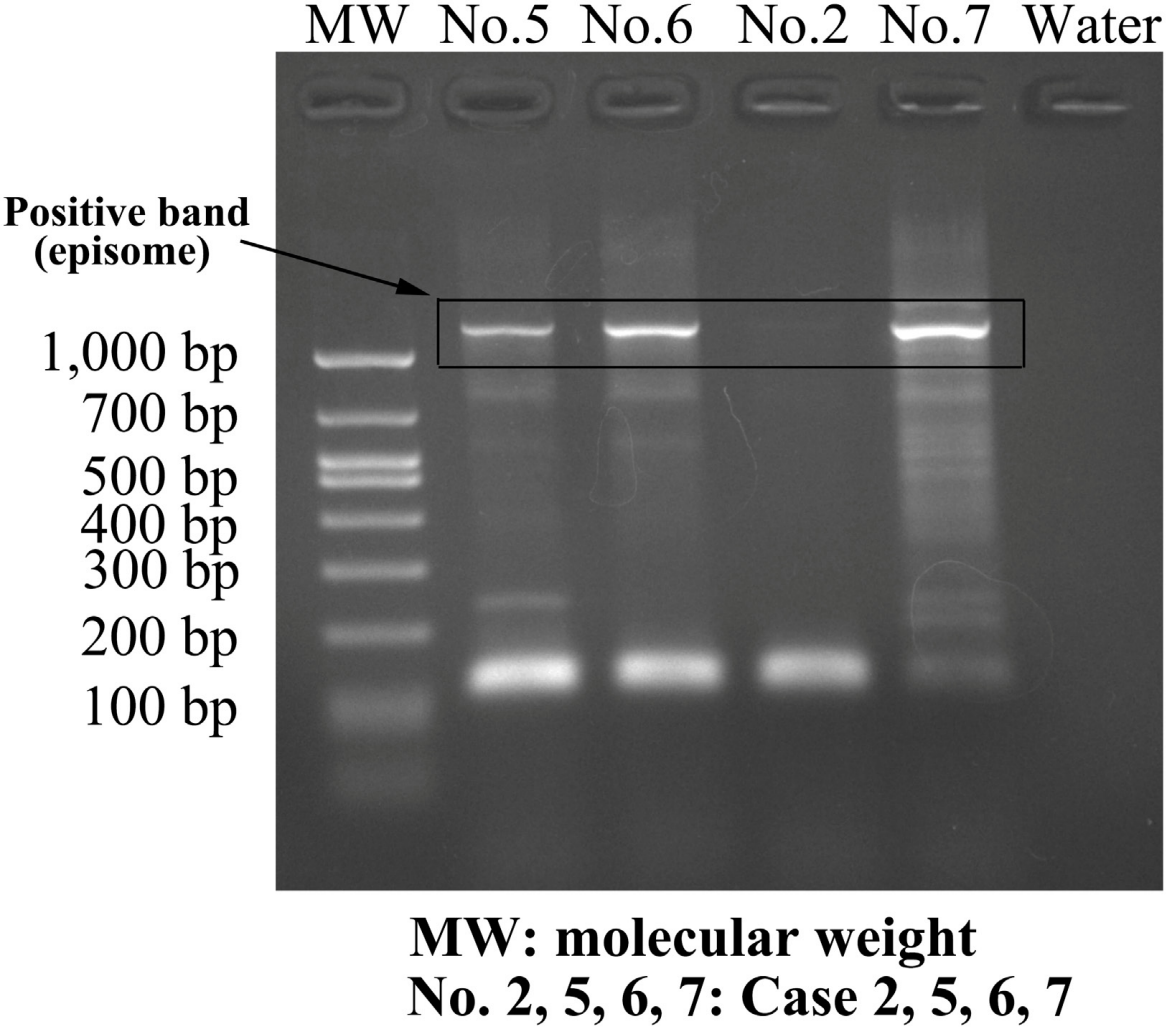
Amplification of papillomavirus oncogene transcripts (APOT) of HPV-6 In several typical cases with HPV-6-positive status, including cases 5, 6, and 7, amplimers of approximately 1200 bp in length were suspected to originate from the E7-E1^^^E4 episomal transcript, and were confirmed by direct sequencing of amplification products. Case 2 was HPV negative.

A 3′-RACE polymerase chain reaction (PCR) that allows differentiation between episome- and integrate-derived *E6/E7* mRNA transcripts was used to determine the physical statuses of the HPV-6 genomes [20]. Regardless of whether the lesions were initial or recurrent lesions, all 15 specimens derived from the 9 HPV-positive patients contained only episomal HPV-6 transcripts with intact *E2*. No integrated-derived *E6/E7* mRNA transcripts were found in these samples (Figure 2).

### Methylation pattern of CpGs at the upstream regulatory region of HPV-6 in AO-LP

**Figure 3:**
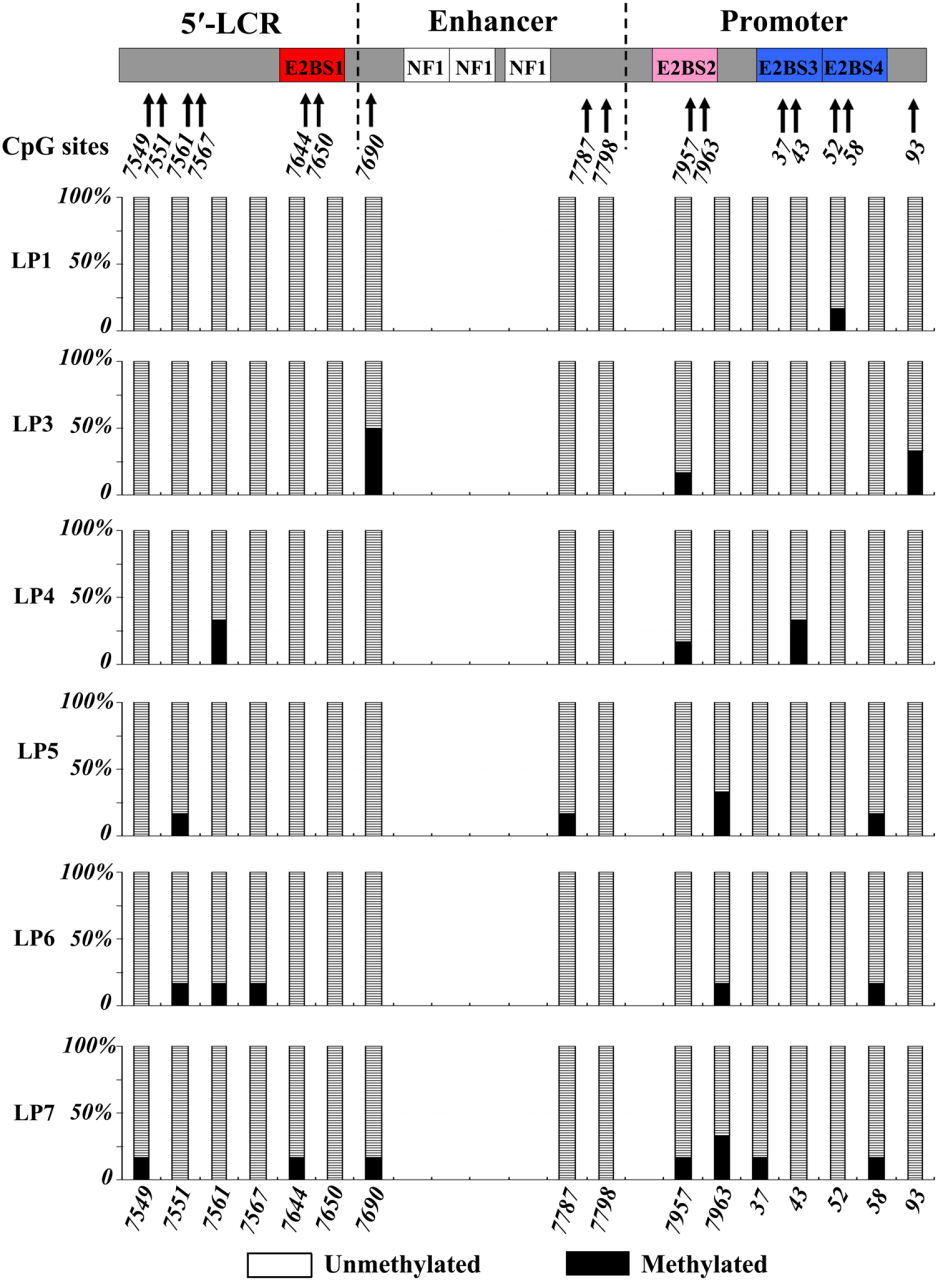
Methylation of CpGs sites at the URR of HPV-6 in AO-LP For every CpG site, 6 clones were sequenced to identify the presence and frequency of meCpG clones. The left vertical bar indicates the percentages of meCpG clones. The structural diagrams of the URR and the positions of the CpG sites are shown at the top and bottom of the figure, respectively. The CpGs in the URR are numbered according to the amended version of HPV6b.

Based on a manual comparison of sequence data from the URR as described by Combrinck et al. [21], HPV-6a, HPV-6b, and HPV-6vc were identified in 2 patients, 2 patients, and 4 patients, respectively (Table 1). The subtype of HPV-6 could not be identified for case 9 due to insufficient tissue. URR sequences and methylation patterns were further analyzed in 10 fresh-frozen specimens collected from 6 of the 11 patients with HPV-6-positive AO-LP, including 6 initial lesions (derived from cases 1 and 3 to 7) and 4 recurrent lesions (cases 5-R1, 5-R2, 6-R1, and 7-R1). Among the 96 CpG sites (16/case), 67 (69.8%) were completely unmethylated, while 23 (30.2%) were heterogeneous (≥ 1 methylated CpG clone). The mean methylation rate in the 6 initial lesions was 5.2% (ranging from 1.0% to 8.3%) for the entire URR region, 3.7% in the 5′-URR, 4.6% in the E6 enhancer, and 6.7% in the p97 promoter. All CpG clones were unmethylated at nt 7650, which is a CpG site of E2BS1, and at nt 7798. At least one CpG clone was methylated at the other CpG sites. Specifically, heterogeneous methylation was found in 50% (3/6) of patients at nt 7953 and nt 7963 of E2BS2, and at nt 58 of E2BS4 within the p97 promoter (Figure 3).

### Expression of p16^INK4a^, p53, and pRb in AO-LP

**Figure 4:**
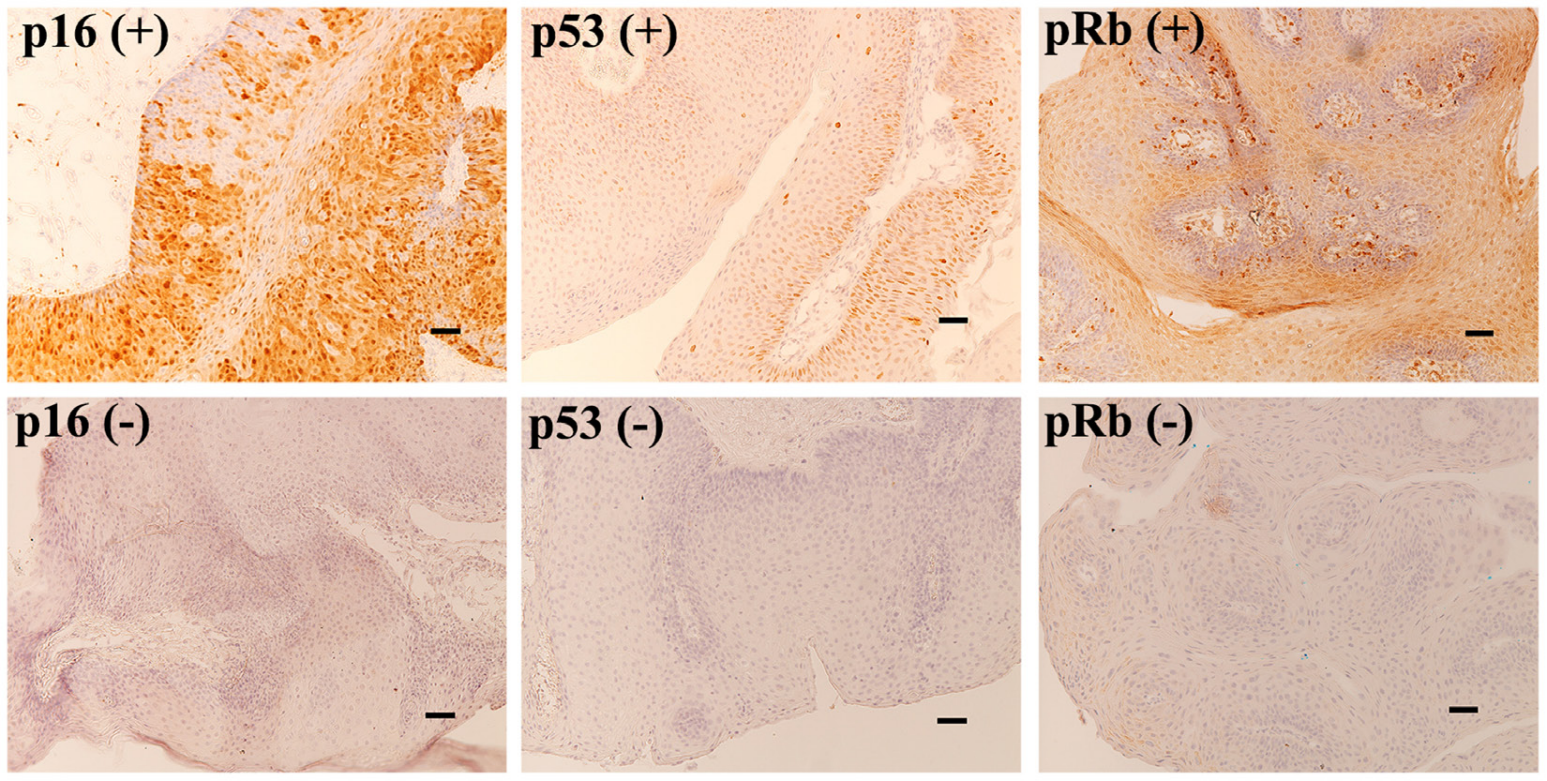
Immunohistochemistry for p16, p53, and pRb in AO-LP Positive expression was defined as p16INK4a staining in > 40%, or p53 and pRb staining in > 25% of 2000 tumor cells. The six micrographs show the typical p16, p53, and pRb immunoreactivity patterns corresponding to positive and negative expression (×100; bar, 100 μm).

The expression of cell cycle proteins (p16^INK4a^, p53, and pRb) in 9 of 11 patients with HPV-positive or -negative AO-LP, including cases 1 to 9, was examined by immunohistochemistry (Figure 4). In the 7 HPV-positive patients, pRb expression was found in 3 cases (42.9%), alongside low percentages of p16^INK4a^ (28.6%, 2/7) and p53 expression (14.3%, 1/7; Table 1). None of the suppressor proteins (p16^INK4a^, p53, and pRb) were expressed in the unique case that developed asynchronous laryngeal papillomatosis and carcinoma.

## DISCUSSION

We studied only HPV-6 because this virus is a unique subtype found in AO-LP populations. The prevalence of HPV-6 (81.8%) was consistent with previous studies, in which 75–80% of the papillomas harbored HPV-6 [22, 23]. Regarding LR-HPV-6 in AO-LP, Forslund et al. measured the *E2/E7* ratio by real-time PCR to determine the physical status of HPV-6 in 25 papilloma samples, and found that laryngeal papillomas and healthy tissue harbored only episomal HPV-6 [24]. A previous study demonstrated that E6 and E7 proteins in LR-HPV are necessary for stable episomal maintenance of LR-HPV genomes [25]. In the present study, we designed two new 5′-primers and developed APOT for HPV-6 based on the method described by Klaes et al. [20], and only the episomal status of virus was identified in all HPV-6-positive AO-LP samples. The persistent expression of *E6* mRNA may contribute to the maintenance of episomal status and replication of HPV-6 in AO-LP.

In a previous study, Huebbers et al. reported a female patient who underwent several hundred revision surgeries for juvenile-onset recurrent respiratory papillomatosis, and eventually developed laryngeal SCC. They identified only the episomal virus in the papilloma and HPV-6 integration in the carcinoma. Loss of the expression of AKR1C3 induced by viral integration may be related to malignant transformation of papillomatosis [26]. In the present study, the patient who developed laryngeal SCC after resection of the papilloma did not harbor HPV-6. Our findings did not support an association between LR-HPV types and the development of laryngeal squamous intraepithelial tumors, and suggest that a carcinogenic mechanism other than HPV-6-related malignant transformation from AO-LP to carcinoma was involved.

To our knowledge, this study is the first to demonstrate dynamic variations in HPV-6 viral loads and mRNA expression among patients with AO-LP. Although no relationship between viral load and recurrence could be established due to the limitation of sample size, our results showed a gradual decline in the tendency of HPV-6 copy numbers and mRNA expression over time after surgical treatment. Both the findings of Forslund et al. [24] and our findings consistently demonstrated large variations in HPV-6 copy numbers among laryngeal papilloma samples. In addition, Forslund et al. reported that the quantity of HPV-6 is not associated with the number of surgical procedures performed since the onset of disease, and they observed significantly higher HPV-6 loads in papillomas compared with paired healthy tissue [24]. Mikolajczak et al. showed that the HPV-6 viral load in AO-LP decreased gradually to zero following several surgeries and intralesional cidofovir therapy. They suggested that relapses can occur if latent laryngeal HPV reservoirs are not eradicated, and HPV replication might recommence from those sites after the end of therapy [22]. Therefore, these findings suggest that the resection range of the lesion should be based not only on pathological diagnosis but also on HPV-6 loads in the adjacent tissues. Persistent infection or residual HPV-6 after treatment may contribute to recurrences of AO-LP. Low-risk E6 and E7 proteins retain many of the same abilities as high-risk proteins that provide redundant mechanisms for promoting cellular proliferation, disrupting apoptosis, and uncoupling cellular differentiation [27]. However, E6 and E7 proteins of LR-HPVs do not seem to express cell-transforming activities comparable with those of their high-risk counterparts [9]. It is known that the ability to promote the degradation of p53 is restricted to high-risk HPV types [8]. In addition, low-risk E7 binds to pRb family members with lower affinity than high-risk E7 [25] and does not target pRb for degradation [9]. Thus, although patients may experience multiple recurrences, tumors rarely undergo malignant transformation.

Previous studies have demonstrated that the methylation of high-risk HPV URR is an important feature in cervical neoplasms, and that it regulates the expression of *E6* and *E7* oncogenes by adjusting the binding of viral E2 protein to the E2BSs [12, 17]. Reuschenbach et al. [28] found that patients with high methylation levels at E2BS3 and E2BS4 had the highest *E6* and *E7* expression levels, and the methylation of E2BS3 and E2BS4 in oropharyngeal SCCs is associated with *E2* integrity and viral physical status. However, there are few studies on the methylation of LR-HPVs in AO-LP. Ure and Forslund reported a lack of methylation in HPV-6 URR from AO-RRP by analyzing 360 clones derived from both basal/intermediate and superficial cells of 6 HPV-6-positive samples. In their study, no methylated CpG was detected in any of the URRs investigated [18]. In the present study, although complete unmethylation was found in most CpG sites of HPV-6 URR, sporadic CpG sites were heterogeneously methylated, including nt 7953 and nt 7963 of E2BS2, and nt 58 of E2BS4. Nevertheless, no correlation between heterogeneous methylation of E2BSs and E6 expression levels in HPV-6-positive AO-LP cases was found. Although the number of samples was limited, our findings provided further evidence of unmethylation in HPV-6 URR and suggested that the methylation of sporadic CpG sites within E2BSs does not sufficiently influence the expression of *E6* and *E7* oncogenes in AO-LP. Moreover, the low frequency of p16^INK4a^ overexpression and low expression of p53 in AO-LP were independent of HPV-6 infection in this study. A previous study by Mooren et al. revealed highly variable staining patterns of p16^INK4a^ in head and neck papillomas and laryngeal dysplasia, irrespective of HPV status [29]. We did not identify an association between recurrence and the expression of p16^INK4A^, p53, and pRb. Further studies of large sample sizes should be conducted to investigate associations between the expression of cell-cycle proteins and recurrence.

In summary, high viral loads and episomal HPV-6 were frequently observed in AO-LP, and persistent *E6/E7* mRNA expression of LR-HPV-6 may be associated with the recurrence of papillomas. Hypomethylation and the scattered patterns of methylated CpGs at the URR of HPV-6 were identified in AO-LP, but our findings did not provide any evidence of malignant transformation from laryngeal papilloma to carcinoma induced by HPV-6. P16^INK4a^ cannot be recommended as a surrogate biomarker for HPV infection in AO-LP.

## MATERIALS AND METHODS

### Ethics statement

The study was approved by the Ethics Committee of University of the Ryukyus, and all participants gave written informed consent. All procedures were conducted in accordance with the ethical standards of the Declaration of Helsinki (1975).

### Study population and specimens

The study included 16 fresh-frozen tissues and 2 formalin-fixed paraffin-embedded (FFPE) specimens obtained from 11 patients with AO-LP who were diagnosed and treated in the Department of Otorhinolaryngology, Head and Neck Surgery, Graduate School of Medicine, University of the Ryukyus, between September 2009 and February 2017.

### Extraction of nucleic acids

Tissue samples were snap-frozen in liquid nitrogen during biopsy or surgical excision and stored in liquid nitrogen for further analysis. A Gentra Purification Tissue Kit (Qiagen, Germantown, MD) was used to isolate DNA from the fresh-frozen samples. The extraction processes were conducted according to the manufacturer’s specifications using the solutions provided in the kit. For the FFPE samples, DNA was extracted by the TaKaRa DEXPAT Easy (TaKaRa, Tokyo, Japan) following the manufacturer’s instructions.

Total RNA was isolated from 5- to 10-mg frozen tumor samples using the ToTALLY RNA kit (Ambion, Austin, TX), according to the manufacturer’s instructions, and suspended in 50 μL ultra-high-quality diethyl pyrocarbonate-treated water. Before cDNA synthesis, any residual DNA was removed through incubation with 1 U DNase I (Ambion) at room temperature for 25 min. cDNA was then synthesized from DNA-free total RNA using the RETROscript Kit (Ambion), according to the manufacturer’s instructions. All assays were performed both with and without reverse transcriptase to examine the presence of contaminating DNA in RNA samples.

### PCR for detecting LR-HPV-6 DNA

The presence of HPV-6 DNA was detected by PCR with two general consensus primer sets, GP5+/GP6+ and MY11/MY09, as described previously [19]. Positive PCR products were purified and directly sequenced, and the sequences obtained were aligned and compared to those of known HPV types in the GenBank database using the BLAST program.

### Viral load and mRNA expression detected by real-time PCR

To detect the viral loads of HPV-6, a quantitative real-time PCR (qRT-PCR) was conducted with the ABI Prism 7300 Sequence Detection System (Applied Biosystems, Carlsbad, CA) and Taqman PCR Master Mix II (Roche Molecular Systems, Foster City, CA). We designed the primers and Taqman probes to target the HPV-6 *E6* open reading frames (Table 2). The *E6* probe was labeled with FAM at the 5′ end and with NFQ at the 3′ end (Applied Biosystems Japan, Tokyo, Japan). Amplification conditions were as follows: 2 min at 50 °C, 10 min at 95 °C, and a two-step cycle of 95 °C for 15 s and 60 °C for 60 s for a total of 50 cycles. A standard curve for the *E6* genes was generated by amplification of serial 10-fold dilutions (10^1^, 10^2^, 10^3^, 10^4^, 10^5^, and 10^6^ viral copies) of a plasmid p1478 carrying the HPV-6b *E6* early region (Addgene, Cambridge, MA). Viral DNA load was determined by calculating E6 copy numbers. Cellular DNA quantification was determined by PCR amplification of β-globin, as described previously [19].

**Table 2:** Primers for real-time PCR, APOT and bisulfite-sequencing PCR for HPV-6

Primers	Sequences (5′ to 3′)
Real-time PCR	
HPV6-RP-forward	GCGTGCTGCCTAGAATTTCAT
HPV6-RP-reverse	CAACAGTTGTTGCATATCCAGCAT
HPV6-probe	FAM-CAAAGTGTCTATATTGGTTAATTTTTC-NFQ
APOT	
HPV6-P1	ACCCTGTAGGGTTACATTGC
HPV6-P2	ACAGCAACGTTCGACTGGTTGTGCAG
HPV6-P3	GACTCGAGTCGACATCG
Bisulfite-sequencing PCR	
Met_HPV6_URR 1F	TWRTTATATTTTGTGATTTAGTGGTTGTTGTA
Met_HPV6_URR 1R	AACACATTATAACAAATTAATAMAAAATATATACYAAAAACA
Met_HPV6_URR 2F	GGTTGTTTTTRGTATATATTTTKTATTAATTTGTTAT
Met_HPV6_URR 2R	AATTAACTACAATACATAAAAATATAACAC
Met_HPV6_URR 3F	GTTTGGTATATAATAATATAAAAATGAGTAATTTAAGGTTATAT
Met_HPV6_URR 3R	TTACAACATATACATAAATAAATTAAACATCTTACACAAC

APOT: amplification of papillomavirus oncogene transcripts

A qRT-PCR with the same E6 primers and probes and the same protocol as that described for the detection of viral load in the previous section was used to estimate cDNA viral load as a measurement of mRNA quantity in the samples, and the quantitative value of *E6* mRNA was described as each value relative to *β-actin* mRNA. The β-actin primers and probes were designed as reported previously [30].

### Detection of HPV-6 physical state by the amplification of papillomavirus oncogene transcripts assay

The 3′ rapid amplification of cDNA ends (RACE) amplification of papillomavirus oncogene transcripts (APOT) assay described previously by Klaes et al. [20] was modified and performed to detect the physical status of HPV-6. We designed and combined a new set of 5′-primers (first 5′-primer, HPV-6-p1; second 5′-primer, HPV-6-p2) with a 3′-Frohman primer (for both nested PCR setups described in Klaes’ study) for nested PCR (Table 2). PCR products of interest were excised from the gel after electrophoresis and extracted using the Wizard Purification Kit (Promega, Madison, WI) for direct sequencing. Sequencing results were analyzed using BLASTN-program provided by the National Cancer Institute (Bethesda, MD).

### HPV-6 methylation analysis

DNA (1–2 μg) extracted from tissue samples was converted with bisulfite using an EpiTect1 Plus Bisulfite Kit (QIAGEN, Valencia, CA), according to the manufacturer’s protocol. The modified DNA was amplified in 3 amplicons with bisulfite-sequencing PCR (BSP) primer assays as BSP-1, BSP-2, and BSP-3 covering the 5′-LCR, and enhancer and promoter regions of the HPV-6 URR, containing 16 CpGs and E2BSs 1, 2, 3, and 4. The primer sequences are shown in Table 2. The 25 μL PCR mixture contained 0.2 mM of each of the 4 dNTPs, 2 mM MgCl_2_, 10 pmol of each primer, 1.25 units AmpliTaq DNA Polymerase (Applied Biosystems) and 300 ng bisulfite-modified DNA. Amplification was carried out under the following conditions: 95 °C for 5 min, followed by 45 cycles at 95 °C for 1 min, 54 °C for 1 min and 72 °C for 2 min, with a final extension at 72 °C for 7 min.

After checking in 2% agarose gel, amplified products were gel purified using a Wizard1 SV Gel and PCR Clean-Up System (Promega) and cloned into the pCR™4-TOPO1 vector (Invitrogen, Carlsbad, CA) for sequencing. For each amplicon, at least 6 individual clones were sequenced using an ABI PRISM 3130xl Genetic Analyzer (Applied Biosystems).

### Immunohistochemistry for p16^INK4a^, p53, and pRb proteins

Serial 4-μm-thick sections from FFPE samples were deparaffinized in xylene and hydrated in a graded series of alcohol. Epitope retrieval was performed by heating at 95–99 °C for 10 min in Tris/EDTA buffer (pH 9.0). Endogenous peroxidase activity was quenched by incubating the sections in 3% hydrogen peroxide and 15 mM sodium azide for 5 min. The sections were subsequently incubated overnight at 4 °C with primary monoclonal mouse anti-p16^INK4a^ antibody (MTM Laboratories AG, Heidelberg, Germany) for p16^INK4a^ staining, primary monoclonal mouse anti-p53 antibody (1:500; Progen Biotech GmbH, Heidelberg, Germany) for p53 staining, and primary monoclonal mouse anti-pRb antibody (1:2000; Life-Span BioSciences, Seattle, WA) for pRb staining. After extensive washing in PBS, the slides were incubated for 30 min at room temperature with a horseradish peroxidase-conjugated goat anti-mouse secondary antibody (MTM Laboratories). Immunolabeling was visualized by incubation in 3-3′-diaminobenzidine and stained slides were counterstained with hematoxylin. Cases were considered pRb-, p53- or p16^INK4a^-positive if intense nuclear and/or cytoplasmic reactivity was present. Positive expression was defined as pRb and p53 staining in more than 25% [31], or p16^INK4a^ staining in 40% of 2000 tumor cells [32].
